# Evaluation of surgical stabilization of metacarpal and phalangeal fractures of hand

**DOI:** 10.4103/0019-5413.33687

**Published:** 2007

**Authors:** Rakesh Gupta, Roop Singh, RC Siwach, SS Sangwan, Narender K Magu, Rahul Diwan

**Affiliations:** Department of Orthopaedic Surgery, Paraplegia and Rehabilitation, Pt. B.D. Sharma, Post Graduate Institute of Medical Sciences, Rohtak - 124 001, Haryana, India

**Keywords:** Metacarpal fractures, phalangeal fractures, surgical stabilization

## Abstract

**Background:**

Optimized functional results are difficult to achieve following hand injuries. This prospective study was undertaken to evaluate the functional outcome after surgical stabilization of metacarpal and phalangeal fractures.

**Materials and Methods:**

Forty-five fractures of digits of hand in 31 patients were managed by surgical stabilization. Five fractures were fixed with closed reduction and percutaneous Kirschner wire fixation; 10 with external fixator; 26 with open reduction and Kirschner wire fixation; and four with open reduction and plate and screw or screw fixation.

**Results:**

Final evaluation of the patients was done at the end of three months. It was based on total active range of motion for digital functional assessment as suggested by the American Society for surgery of hand. Overall results were excellent to good in 87%. Better total active range of motion (excellent grade) was observed in metacarpal fractures (47%) versus phalanx fractures (31%); closed fractures (57%) versus open fractures (27%); and single digit involvement (55%) versus multiple digits (29%). Excellent total active range of motion was observed with all four plate and screw/ screw fixation technique (100%) and closed reduction and percutaneous kirschner wire fixation (60%). Twenty-two complications were observed in 10 patients with finger stiffness being the most common.

**Conclusion:**

Surgical stabilization of metacarpal and phalangeal fractures of hand seems to give good functional outcome. Closed fractures and fractures with single digit involvement have shown a better grade of total active range of motion.

Fractures of metacarpals and phalanges constitute between 14-28% of all visits to the emergency department.^1^ Functional outcome of the fractures of small bones of the hand is partly dependent upon the severity of initial injury and its management.^2^ Fracture healing in the hand is not an isolated goal; rather, the functional end result is of paramount importance.^3^ The purpose of this prospective study was to evaluate operative results of metacarpal and phalangeal fractures.

## MATERIALS AND METHODS

Forty-five metacarpal and phalangeal fractures of the hand in 31 patients aged 14 yrs or more were included in the prospective study conducted during the period 2004-2005. Unstable metacarpal and phalangeal fractures, intraarticular fractures, avulsion fractures, fracture dislocations and open fractures with sharp and clean wounds were included in the study. An unstable fracture was defined as one in which the patient was able to move the adjacent joints by less than 30% of the expected normal range of motion.^2^ Open fractures with severe soft tissue injury and fractures associated with severe osteoporosis were excluded.

The fractures were reduced and fixed using various implants including Kirschner wires, external fixators, plates or screws depending on fracture site, configuration and associated soft tissue damage. The fractures were divided into four groups depending on the type of internal fixation.

Group I (n=5): The closed reduction and percutaneous Kirschner wire fixation was done in patients with fractures of the middle phalanx, proximal phalanx and metacarpal when closed reduction was possible.

Group II (n=10): Open/closed reduction and external fixation was performed for open fractures with sharp and clean wounds.

**Figure 1:**
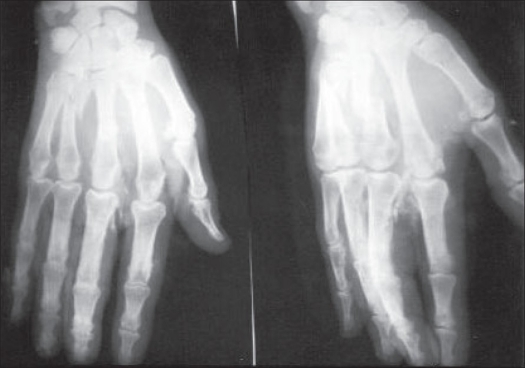
Preoperative radiographs showing open fractures of III^rd^ and IV^th^ metacarpal

**Figure 2:**
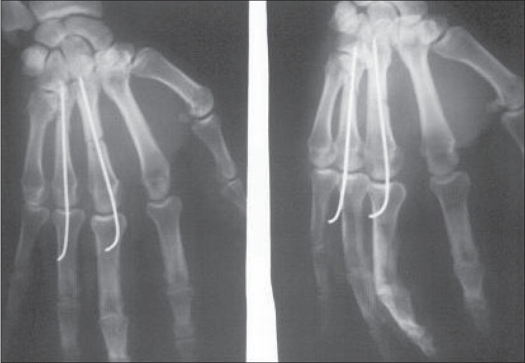
Postoperative radiographs showing good alignment after fixation with two Kirschner wires

**Figure 3:**
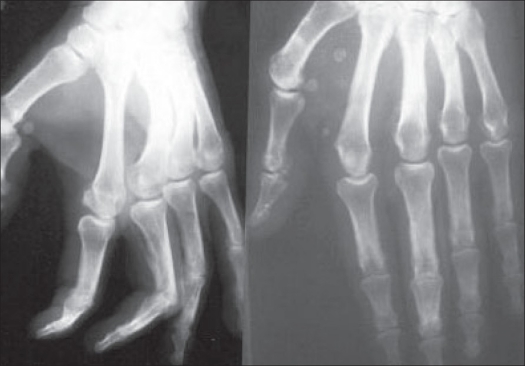
Radiographs after Kirschner wire removal showing union of fracture and good alignment

**Figure 4:**
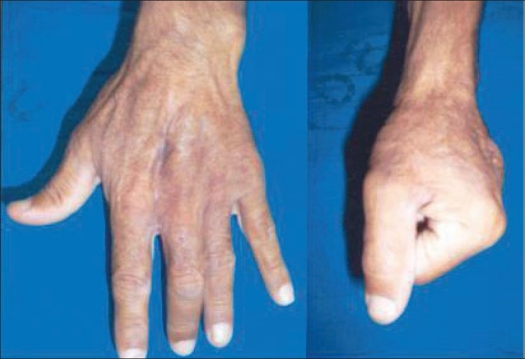
Clinical photographs showing excellent total active range of motion at follow-up of six months

Group III (n=26): Open reduction and Kirschner wire fixation was done in fractures where closed reduction was not possible [Figures 1–4]. Retrograde Kirschner wire fixation, intramedullary Kirschner wire fixation avoiding the joint and cross Kirschner wire fixation was done in 16, six and four fractures respectively.

**Figure 5:**
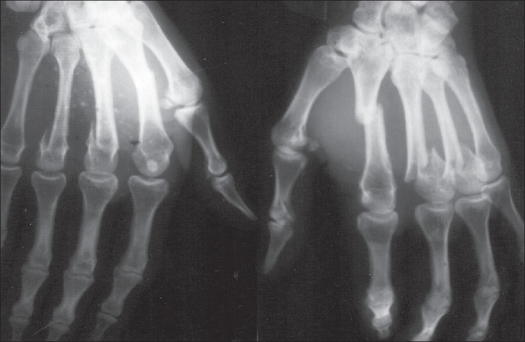
Preoperative radiographs showing transverse fracture of 2nd metacarpal; and fracture neck of 3^rd^ and 4^th^ metacarpals

**Figure 6:**
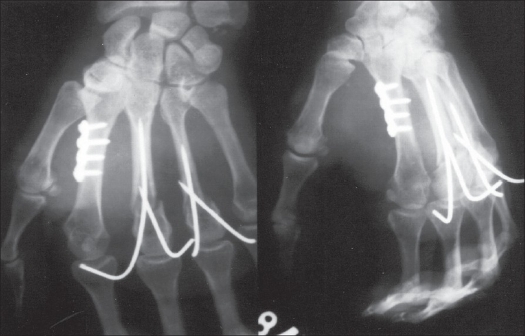
Postoperative radiographs showing good fixation with plate and screws of 2nd metacarpal fracture; and fixation with Kirschner wires for fracture neck of 3^rd^ and 4^th^ metacarpals.

**Figure 7:**
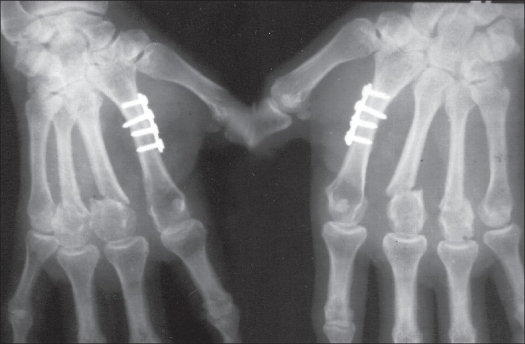
Radiographs showing union of fractures after removal of Kirschner wires

**Figure 8:**
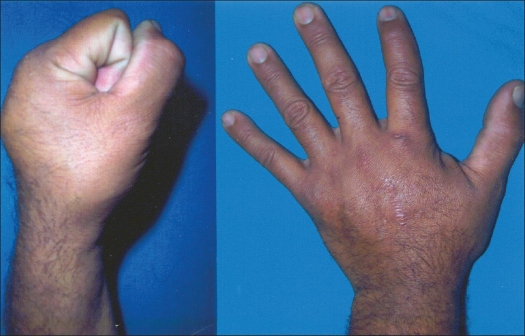
Clinical photographs showing excellent hand function at follow up of six months.

Group IV (n=4): Here open reduction with plate and/or screw fixation was performed for long oblique or spiral fractures of both metacarpals and phalanges [Figures 5–8]. The avulsion fractures of the proximal phalanx were fixed with screw alone in two fractures; while plate fixation was used for transverse and short oblique fractures of metacarpals in the remaining two.

The hand was kept elevated and patients were encouraged to perform movements of the fingers and hand to prevent edema and stiffness. Antibiotics were given for 48h and prolonged in cases of open fractures. The Kirschner wires and external fixation devices were removed between 3 to 6 weeks. Active assisted mobilization was started after removal of Kirschner wire / external fixator usually at three to six weeks. Wax bath and contrast bath were given. Continuous passive motion (CPM) was not used. Patients were followed weekly for the first month and fortnightly for the next two months before final evaluation at three months.

## RESULTS

The mean age of the patients was 35.6 years (range 14-74 years). The most common mode of trauma was assault and roadside accidents (60%). In all, 43 digits were involved and there were a total of 45 fractures. Twenty-two patients had only a single digit involvement while two digits were involved in six patients and three digits in three patients. Fracture site and configuration in metacarpal and phalanges is shown in Table 1. Nine hands had more than one fracture and multiple metacarpal fractures were the most common combination. Twenty-three were closed and 22 were open fractures. Articular involvement was observed in nine fractures.

**Table 1:** Fracture site and configuration

Site		Configuration	Number of fractures	Percentage
Distal phalanx	Shaft	Transverse	1	2.22
Middle phalanx	Shaft	Transverse	1	2.22
		Comminuted	1	2.22
Proximal	Neck	Transverse	1	2.22
phalanx	Shaft	Transverse	4	8.89
		Oblique	2	4.44
	Base	Comminuted	2	4.44
		Avulsion	1	2.22
Metacarpal	Head with			
	MP joint			
	dislocation	Avulsion	2	4.44
	Neck	Transverse	4	8.89
	Shaft	Transverse	13	28.89
		Oblique/ Spiral	7	15.57
		Comminuted	1	2.22
	Base	Avulsion	1	2.22
		Comminuted	3	6.68
		Oblique	1	2.22
Total			45	100

The functional outcome after fracture treatment was assessed by calculating total active range of motion (TAM).^4^ This was done by adding the active flexion at metacarpophalangeal, proximal interphalangeal and distal interphalangeal joints, after subtracting the sum of extension deficit at these three joints. Recovery is calculated as percent-regained motion compared to normal range of digital motion (260°). According to this patients with 85-100% of movement are classified as excellent; 70-84% as good; 50-69% as fair; and < 50% as poor.

Metacarpal fractures (n=32) had an excellent (n=15, 47%); and good (n=15, 47%) TAM in 94% of fractures [Figures 1–4], whereas only 54% (excellent: n= 4, 31%; good n=3, 23%) of phalangeal fractures (n=13) achieved this range of motion. Closed fractures (n=23) had a better end result with excellent (n=13, 57%) and good TAM (n=9, 39%) as compared to open fractures (n=22) with excellent (n=6, 27%); good (n=9, 41%). A higher TAM was observed with single digit involvement (n=12) as compared to fractures that involved more than one digit (n=6). Final TAM was not related to the fracture configuration present in the digit.

Overall end results of hand fractures in 31 patients managed by surgical stabilization were excellent in 14 (45.16%); good in 13 (41.93%); fair in three (9.68%); and poor in one(3.23%).

A total of 22 complications were observed [Table 2] in 10 patients out of a total of 31. Finger stiffness (15.56%) and deformity (7%) were the most commonly observed complications.

**Table 2:** Complications observed in the present series

Complications	Metacarpal fractures	Phalangeal fracture	Total	Percentage (out of 45 fractures)
Finger stiffness	2	5	7	15.56
Infection	0	2	2	4.44
Kirschner wire/ pin				
loosening	1	0	1	2.22
Skin necrosis	1	0	1	2.22
Pin tract infection	0	1	1	2.22
Deformity	2	1	3	6.67
Nonunion	1	1	2	4.44
Malunion	1	1	2	4.44
Articular incongruity	1	1	2	4.44
Finger clawing	0	1	1	2.22
Total	9	13	22	

Results in various groups according to TAM are tabulated in Table 3. Best results were observed in fractures treated with open reduction with plate and / or screw fixation (Group IV) [Figures 5–8]. The statistical difference could not be drawn between different groups, as patients in some groups were very less.

**Table 3:** Results in various groups according to total active range of motion

Group	Total fractures	Total active range of motion			
		
		Excellent (%)	Good (%)	Fair (%)	Poor (%)
I	5	3(60.00)	1 (20.00)	1 (20.00)	0 (0.00)
II	10	2 (20.00)	4 (40.00)	3 (30.00)	1 (10.00)
III	26	10 (38.46)	14 (53.85)	2 (7.69)	0 (0.00)
IV	4	4 (100.00)	0 (0.00)	0 (0.00)	0 (0.00)

## DISCUSSION

Many factors, such as delicate handling of tissues, preservation of gliding planes for tendons, prevention of infection and early and appropriate physiotherapy other than accurate reduction and fixation affect recovery of good mobility.^5^

A higher incidence of excellent TAM (n=13, 57%) was observed in closed fractures as compared to open fractures (n=6, 27%). Pun *et al.*^6^ (n=235) have also reported a lower grade of TAM in open fractures (42% good result) as compared to closed fractures with 70% good results. Page and Stern^7^ (n=82) in their study reported excellent TAM in 67% of closed fractures with only 24% in open fractures. Tan *et al.*^8^ (n=28) also observed that open fractures had poor final TAM as compared to closed fractures; although all the cases were intraarticular in that series.

Closed reduction and percutaneous Kirschner wire fixation was done where fracture could be reduced and maintained by closed means. Of the five fractures treated, three achieved excellent TAM (60%). Belskey *et al.*^9^ (n=100 proximal phalanx fractures) and Green and Anderson^10^ (n=26) reported similar results. Although, the number of fractures in the present series is less to derive any definite conclusion regarding superiority of the procedure.

External fixator was used for surgical stabilization of 10 open fractures. Advantage of external fixation is that no joint transfixation is required and hence patient does not develop any stiffness of the adjacent joints. Sixty per cent (n=6) of the fractures treated by this technique achieved excellent to good TAM. The results are comparable to those reported by Freeland^11^ (n=12) (70% excellent to good TAM). However, the results of the present study are inferior to those obtained by Schuind *et al.*^12^ (n=63) who reported 96% excellent to good TAM. This is probably on account of the fact that the study of Schuind *et al.*^12^ included only closed fractures whereas external fixation was used for only open fractures in the present study and final outcome is definitely expected to be compromised to some extent in open injuries.

Open reduction with Kirschner wire fixation was done for stabilization of 26 fractures using three different techniques. (a) Retrograde insertion of Kirschner wire (n=16) with transfixation of joint was first advocated by Vomsaal.^13^ No significant stiffness was observed in cases of metacarpal fractures treated by this technique when metacarpophalangeal joints were fixed in flexion, while one proximal phalangeal fracture developed extension lag when proximal interphalangeal joint was fixed in flexion. (b) Intramedullary Kirschner wire without transfixing the joint (n=6) is technically more difficult than retrograde Kirschner wire fixation. No stiffness was observed in any of the cases treated by this method. (c) Cross Kirschner wire fixation using two Kirschner wires was done for stabilization of four fractures. The advantage of this technique is that it does not permit rotation of fracture fragments, thus making early mobilization possible, as compared to the technique of intramedullary fixation where chances of rotation of fragments do exist. However, Ikuta and Tsuge^14^ reportedly observed distraction with crossed Kirschner wire fixation using two wires, thereby holding it responsible for delayed union and nonunion. However, no nonunion or delayed union was observed by us following cross wire fixation with two Kirschner wires. It is desirable that while using cross Kirschner wire fixation crossing point of the wires should not be located at the fracture site so as to avoid distraction.

Open reduction and plate fixation was done in two fractures and two patients were treated with screw fixation. Excellent TAM was obtained in all four fractures treated by these techniques. Bosscha and Snellen^15^ (n=43) achieved 92% excellent TAM. Page and Stern^7^ (n=82) have, however, reported a high incidence of complications with plate fixation, especially in phalangeal fractures. We observed no complications. The number of cases undergoing plate fixation was too small and no plate fixation was done in phalangeal fractures. Open reduction with screw fixation was done for stabilization of two fractures. Both the cases achieved excellent TAM. Although, the number of fractures in the present series is less to derive any definite conclusion regarding the superiority of the procedure. Only a single screw was used in both the cases. Crawford^16^ observed that although screw fixation was excellent for proximal phalangeal fractures it did not offer much advantage over conservative means for similar metacarpal fractures. This is disputed by Dabezies and Schutte^17^ who advocated screw fixation for spiral fractures of metacarpals, as these are prone to rotational deformities. Shewring and Thomas^18^ have also recommended surgical stabilization of avulsion fractures with a screw, as the fractures which were not stabilized went into nonunion.

Finger stiffness was the most commonly observed complication in the present series. Only one case of open fracture developed superficial infection. None of the cases developed osteomyelitis. Two patients with multiple fractures developed angulation at fracture site as the Kirschner wire was removed four weeks postoperatively before any radiological union. The movements, however, were almost full at all joints. Two cases of multiple fractures developed hypertrophic nonunion after Kirschner wire fixation. The cause was thought to be inadequacy of Kirschner wire fixation, which cannot check movements at fracture site completely. Ikuta and Tsuge^14^ have also blamed improper Kirschner wire fixation for nonunion.

The results of the present study following surgical stabilization of fractures of metacarpals (94% excellent to good) and phalanges (54% excellent to good) were observed to be superior to those reported by Duncan *et al.*^19^ (63% excellent to good for metacarpal fractures and 32% excellent to good for phalangeal fractures). Most of the authors have made similar observations indicating superior TAM following metacarpal fractures as compared to phalangeal fractures [Table 4].

**Table 4:** Comparison of total active range of motion in different series

Series	Excellent %		Good %		Fair %		Poor %	
				
	Metacarpal	Phalangeal	Metacarpal	Phalangeal	Metacarpal	Phalangeal	Metacarpal	Phalangeal
Page and stern^7^	62	8	14	3	13	27	11	62
Schuind *et al.*^12^	86.4	62.7	10.2	31.4	-	-	3.4	5.9
Stern *et al.*^20^	50	33.3	37.5	33.4	-	-	12.5	33.3
Duncan *et al.*^19^	40	16.12	24.6	14.5	7.7	12.9	27.7	56.5
Drenth and klasen^2^	34.48	71.4	34.48	-	10.4	-	20.7	28.6
Shehadi^21^	100	98	77	66	-	-	-	-
Present study	46.86	30.77	46.88	23.08	6.24	38.46	0	7.69

Figures all in percentage

Limitation of the study: The sample size is very small in some groups for statistical comparison.

## CONCLUSION

Surgical stabilization of metacarpal and phalangeal fractures of the hand seems to give good functional outcome. Closed fractures and fractures with single digit involvement are important determinants to achieve a better grade of total active range of motion.
